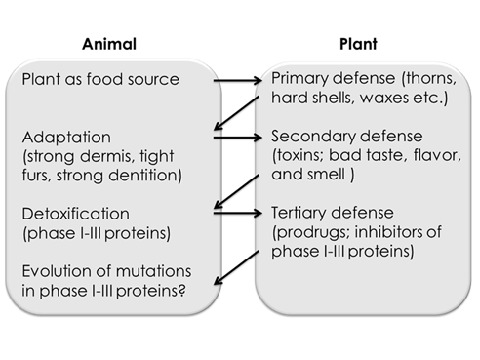# Animal plant warfare and secondary metabolite evolution

**DOI:** 10.1007/s13659-013-0004-0

**Published:** 2013-03-08

**Authors:** Steffen Wöll, Sun Hee Kim, Henry Johannes Greten, Thomas Efferth

**Affiliations:** 14 Department of Pharmaceutical Biology, Institute of Pharmacy and Biochemistry, Johannes Gutenberg-University, Mainz, Germany; 24 Heidelberg School of Chinese Medicine, Karlsruher Straße 12, 69126 Heidelberg, Germany; 34 Biomedical Sciences Institute Abel Salazar, University of Porto, Porto, Portugal

**Keywords:** natural products, phytochemicals, liver metabolism, cytochrome P450 monooxigenase, ABC-transporter, herbivore

## Abstract

**Abstract:**

The long-lasting discussion, why plants produce secondary metabolites, which are pharmacologically and toxicologically active towards mammals traces back to the eminent role of medicinal plants in the millennia-old history of manhood. In recent years, the concept of an animal plant warfare emerged, which focused on the co-evolution between plants and herbivores. As a reaction to herbivory, plants developed mechanical defenses such as thorns and hard shells, which paved the way for adapted animal physiques. Plants evolved further defense systems by producing chemicals that exert toxic effects on the animals that ingest them. As a result of this selective pressure, animals developed special enzymes, *e.g*. cytochrome P450 monooxigenases (CYP450) that metabolize xenobiotic phytochemicals. As a next step in the evolutionary competition between plants and animals, plants evolved to produce non-toxic pro-drugs, which become toxic only after ingestion by animals through metabolization by enzymes such as CYP450. Because these sequestered evolutionary developments call to mind an arms race, the term animal plant warfare has been coined. The evolutionary competition between plants and animals may help to better understand the modes of action of medicinal plants and to foster the efficient and safe use of phytotherapy nowadays.

**Graphical abstract:**

## 1 Plant Defense Systems Against Herbivores

The relevance of animal-plant warfare can be illustrated by an apparent example from every-day life: Anyone who has ever cut an onion while cooking knows how effectively plants can cause reactions: tearing and burning eyes may prevent cooks or animals from destroying *Allium sativum* bulbs. With the evolution of this defense mechanism, the plant has won an important fight against its enemies. Plants were challenged during evolution by microbial attack and herbivores. Lacking an immune system such as animals and limited control over their movements, they developed a chemical defense system with effective toxins.[1] The field of chemical ecology has emerged during the past two decades and a lot of interactions between plants and hervivores have been understood. It has been a matter of debate for many decades that evolutionary selection leads to optimization of survival strategies and adaptation of species on earth (adaptionist programme).[2]

Plants have evolved several mechanisms against external attacks. Constitutively expressed defenses offer plants permanent protection, while inducible defenses are produced only in response to environmental stresses such as mammalian browsing and insect herbivory.[3,4] The variability of environments, including attributes such as altitude, temperature and moisture variations, are further factors influencing plants' armory of defensive weapons.[5,6]

The various phytochemicals produced by plants are major constraints that limit the ingestion behavior of herbivores and thus determine herbivore impact on vegetation. Plant secondary metabolites (PSMs) such as alkaloids, terpenes, phenolics, glucosinolates and cyanogenic glycosides have been shown to exert effects on herbivores.[7] The biosynthesis of these compounds is regulated by genes whose expression and activity are directly influenced by environmental factors. In this context, the influence of fire on the coevolution of plants and herbivores has been pointed out.[8] Fire is a repeatedly occurring disturbance for flora and fauna in some biosystems. It has been shown that where fire is greatest, hares are most abundant. They feed on juvenile woody plants when vegetation grows again after destruction by fire. As an adaptation, birches (*Betula neoalaskana* and *B. papyrifera*) produce more PSM in these regions than birches in areas without fire. Hence, geographic conditions such as bush fire coincidences with coevolution of plants and herbivores.[8]

The concentrations of specific hydrocarbons, fatty acids and essential oils that a plant produces also depend on other environmental conditions.[3,9] Increased concentrations of toxic compounds may cause directly serious health consequences in herbivores. Hence, herbivores must avoid saturating their detoxification systems as stated in the detoxification limitation

*To whom correspondence should be addressed. E-mail: efferth@uni-mainz.de hypothesis.[10–12]

PSM of food plants restrict the amount of food taken up by animals and thereby affect their reproduction rates.[13] Hence, PSM reduce fitness of herbivoric predators.[13,14] During evolutionary development, herbivores reacted by different strategies, *e.g*. development of sophisticated oral sensation of the PSM content in a plant[15] reduced food intake[16] switching from diet with high PSM content to another one with less concentrations of toxins,[17,18] alterations of meal patterns, *i.e*. changing from few daily meals with high food intake to many meals with low food intake,[19,20] and metabolization and detoxification of toxic PSM taken up with food.[21–25] Another adaptation to PSM that cannot be taken up in unlimited amounts is the evolutionary developments of specialists and generalists. The mixed diet of generalistic herbivores reduce the need for detoxification of similar groups of toxic PSM, whereas herbivores specialist to one or few plant species as food source developed especially improved metabolic detoxification pathways.[26–31]

In the past decades, many phytochemicals have been isolated and investigated for their pharmacological activities. Examples include Lamiaceae plants such as lavender (*Lavandula angustifolia* Mille. *x L. latifolia* Mill.), thyme (*Thymus vulgaris* L.), winter savory (*Satureja montana* L.), rosemary (*Rosmarinus officinalis* L.), sage (*Salvia officinalis* L.) and peppermint (*Mentha x piperita* L.), all of which contain chemical compounds to protect against herbivores (Table 1; Figure 1).[32]

Mammalian herbivores that ingest plants may suffer from a variety of adverse effects. It has been reported that PSMs stimulate the emetic center so that herbivores increasingly deter these plants by flavor aversion.[33] Two specific monoterpenoids of thyme basil (*Acinos suaveolens*, Lamiaceae), *R*-(+)-pulegone (cyclohexanone, 5-methyl-2-(1-methylethylidene) and menthone (cyclohexanone, 5-methyl-2-(1-methylethyl)), have been analyzed and provide several interesting clues supporting the hypothesis of evolutionary animal plant warfare. In a study of plants in Greece, significant differences in the concentration of these compounds in upper, middle and lower sections of plants on accessible and inaccessible rock pillars were found.[34] After an extracting process, the levels of menthone and pulegone were determined by gas chromatography coupled with mass spectrometry (GC-MS). The concentrations of menthone were similar, whereas plants in the accessible rock location contained more *R*-(+)-pulegone than plants on inaccessible rock pillars. Previous experiences have shown that invertebrates avoid *R*-(+)-pulegone-containing Lamiaceae plants. These two monoterpenoids possess antimicrobial, fungistatic, allelopathic, insecticidal,[35] and even psychoactive properties.[36] Pulegone seems to be more toxic than menthone to herbivores. Symptoms such as necrosis, pulmonary edema and internal hemorrhage appear after consumption of pulegone.[37] It has been hypothesized that monoterpenoids covalently bind to liver proteins leading to microsomal cytochrome P450 damage.[38,39] Consequently, xenobiotics are unable to be metabolized due to liver dysfunction.

The toxicity of pulegone was demonstrated in a study on pennyroyal tea used against various ailments and as an arbotifacient.[40] This tea may lead to death in some cases because of the high toxicity of pulegone. It has traditionally been used to settle an upset stomach, to relieve flatulence, to induce sweating, and to treat colds, influenza and abdominal cramps. It is also applied for the treatment of diseases such as smallpox and tuberculosis and to promote latent menstruation.

Biochemically, pulegone is a precursor of menthone that is synthesized by pulegone reductase.[41–43] Genetic modification of this enzyme leads to differential processing of chemical compounds.[42–45] It can be hypothesized that plants easily accessible to herbivores, such as those in meadows, reduce the expression of pulegone reductase so that the *R*-(+)-pulegone levels are increased. On the other hand, plants on inaccessible rock pillars produce less pulegone. Similar inter-individual variability as an adaptation against herbivore attack has been observed for other terpenoid compounds as well.[46,47]

Another example of animal plant warfare is presented by *Mintosthachys mollis* (Lamiaceae), a plant used for medicinal infusions and for the preparation of commercial drinks due to its peppermint flavoring. It is also a natural biocide used to control pests.[44] These plants face two different types of insect herbivores: the leaf miner *Chromatomyia platensis* (Brethes) (Diptera: Agromyzidae) and the cecydomid gall insect (Diptera: Cecydomyiidae).[48–50] The concentration of terpenoids in different parts of the plant affected by leaf miner insect and undamaged leaves as well as leaves from stems with and without galls was determined. The monoterpenes pulegone and menthone accounted for about 70%.[44,51] The leaves that had been mined or were growing on gall-bearing stems revealed significantly decreased concentrations, whereas both categories of undamaged leaves (mined or were growing on gall-bearing stems had similar concentrations of pulegone. This speaks to a chemical defense reaction against insect-induced galls and other lesions. Furthermore, the leaves growing on a gall-bearing stem showed increased menthone

**Table 1. Important chemical compounds in the Lamiaceae family of plants**

| plant | chemical compounds |
|---|---|
| *Lavandula angustifolia* Mille. x L. latifolia Mill. (lavender) | 1,8-cineole, linalool, camphor and linalyl acetate |
| *Thymus vulgaris* L. (thyme) | *p*-cymene, *τ*-terpinene and thymol |
| *Satureja montana* L. (winter savory) | *p*-cymene, *γ*-terpinene and carvacrol |
| *Rosmarinus officinalis* L. (rosemary) | *α*-pinene, borneol, 1,8-cineole and camphor |
| *Salvia officinalis* L. (sage) | 1,8-cineole, *α*-thujone, *β*-thujone and camphor |
| *Mentha x piperita* L. (peppermint) | 1,8-cineole, menthone, menthofuran, menthol and pulegone |

**Figure 1.** Chemical structures of selected natural products (taken from Wikipedia)

concentration. The accelerated rate of pulegone transformation to menthone was understood to be a response to leaf miner activity. Similar results were obtained in the presence of galls, although other mechanisms were hypothesized. All chemical compounds in *Mintosthachys mollis* were generally recognized as safe by the U.S. Food and Drug Administration.[52]

These two examples of thyme basil and *Mintosthachys mollis* illustrate the way in which plants respond to animal herbivores.[53] Mammalian herbivore attacks lead to an increase in pulegone concentrations, whereas the content of menthone is generally increased when insects attack plants.

The first plant barriers are the cell wall and the cuticle.[6] Epicuticular waxes,[6] although they primary protect from fluid loss and high temperatures, also double as important plant barriers against external attacks. Another anti-herbivore defense mechanism is the development of thorns, spines and prickles.[54] Many types of thorns are aposematic meaning that they are colored, characterized by bright conspicuous markings, which mammalians recognize and learn to avoid. Mammalian herbivores learn to recognize and avoid the distinctive coloration of specific plant parts after repeated injury or adverse reaction.

## 2 Evolutionary Arms Race Between Plants and Herbivores

Plants use three major levels of strategic defense to protect themselves from herbivores (Figure 2). Firstly, physical protection consists of thorns, spines and strengthened cell walls, which cause ligneous, hard shelled bodies that deter animals from galling.[7] Secondly, PSMs provide chemical protection. PSMs can cause herbivore aversion by bitter taste, bad flavor or smell. Furthermore, PSMs can be toxic and induce vomiting, illness or dizziness in animals after plant consumption. The third defense strategy represents metabolic defenses. Many PSMs act as pro-drugs in animals, *i.e.* they need to be metabolized by CYP450 enzymes to deploy their toxic effects in the body. Some plants are able to reduce the effectiveness of PSM detoxification by inhibiting efflux pumps (phase III proteins).[55] Efflux transporters have initially raised interest in cancer biology and clinical oncology as pumps for anticancer drugs that maintain the level of anticancer drugs at sublethal concentration in cancer cells thereby leading to threatened multidrug resistance phenotypes failure of chemotherapy and fatal outcome for cancer patients.[56–59] More recently, it was recognized that efflux transporters play a crucial role for the absorption and bioavailability of pharmaceuticals in general and xenobiotic compounds taken up with food.[12,60,61] This also implies a fundamental role of the evolutionary arms race between animals and plants.

On the other hand, animals defend themselves against these plant attacks at several strategic levels. Analogous to the physical plant defenses, animals developed robust physical attributes such as strong dermis, tight furs or coat, strengthened dentition, *etc*. At the secondary stage, animals developed protective strategies against the chemical toxins produced by plants. This includes specific feeding habits to avoid toxic plasma levels as well as the development of detoxifying mechanisms.[7] In detail, there are four steps in plant chemical toxicity that can theoretically be countered by mutations. The uptake of xenobiotics into a cell is described as "Phase 0", and of course it is in the animals' best interest to prevent the diffusion or uptake of toxic compounds. "Phase I" comprises the metabolism of xenobiotics via CYP450 enzymes. Adaptation in the enzymes' structure or in their induction leads to increased detoxifying potential. This detoxification step represents the single most effective way for animals to

**Figure 2.** The animal plant warfare system as a mutual correlation cope with toxic influences from their environment. In "Phase II," the phase I metabolites are coupled with hydrophilic substances to increase elimination of toxins. Increased elimination results in an ability to tolerate higher amounts of toxin uptake because it decreases plasma toxin levels.[7] Therefore, mutations in phase II enzymes that boost their effectiveness towards a certain xenobiotic may entail nutritional advantages. The forth step that poses an opportunity for evolutionary adaptation is the "phase III" detoxifying mechanism that consists of transporter proteins. Phase III proteins include multidrug resistance proteins (MDRs), organic anion transporters (OATs) and organic cation transporters (OCTs).

The warfare between possums and *Eucalyptus* plants is an illuminating example of an effective animal detoxification system. Possums ingest doses of up to 10 g of 1,8-cineole per day, doses which would be quite poisonous for human beings. Terpenes act as inducers of their own metabolism by positive feedback.[55] Animals which have been fed ten days with a terpene-diet showed a 53% increase in CYP450 enzymes. The terpenes are metabolized into 19 different metabolites, which are excreted in free or glucuronidated form.[62] On the other hand, *Eucalyptus* trees have reduced their attractiveness for herbivores: Beside their high content of toxic oils, they contain only low amounts of protein and high lignin fiber.

PSM influence on gene expression has been observed in many other plants. A prominent representative is St. John's Wort, whose constituent ingredients increase CYP3A4 and P-glycoprotein (*MDR1*) levels in herbivores.[63] Another interesting point is that chlorophyll is an inducer of phase II enzymes,[64] which are responsible for improved elimination of transformed xenobiotics. This up-regulation of enzymes supporting the excretion of toxins led to the hypothesis of a reversal effect: animals may use certain PSMs for protection against other toxic plant compounds.

The evolution of natural pro-drugs can be viewed as a result of improved detoxifying systems in animals. The effectiveness of CYP450 to dispose the plants' toxins made it necessary to develop strategies which are able to skirt the animals' defense. These so called pro-drugs allow plants to co-opt the animal detoxification system to increase the effectiveness of their own toxins and therefore their chance of survival. The underlying principle is that the plants use these detoxifying enzymes as tool to activate non-toxic PSMs into toxic metabolites within animal cells. Taking into account that the main detoxification organ is the liver, hepatotoxicity is a frequent consequence of metabolism of these pro-drugs.[65]

### 3 Metabolic Detoxification Systems of Animals

Because of the extraordinary importance of metabolic detoxification of PSM in animals, we focus in more detail on the large superfamily of cytochrome P450 (CYP450) enzymes and their role in plant-herbivore interaction. Present in almost all eukaryotes and prokaryotes, the functional spectrum of CYP450 enzymes is much broader than simply detoxifying exogenous plant compounds. In fact, CYP450 enzymes are even involved in the synthesis of PSMs in plants, as well as in the conversion of other endogenous substrates such as hormones or vitamin $D_3$, and in basic cellular functions such as cell growth and apoptosis.[66]

The CYP450 enzymes are a family of heme-thiolate proteins with a characteristic absorption at 450nm while they bind a CO-molecule as the sixth ligand in a characteristic low-spin heme-thiolate-iron-complex. More than 2000 members of this superfamily of enzymes have been identified.

The CYP450 enzymes are essential for elimination of exogenous xenobiotic compounds. Therefore, the highest expression of CYP450 enzymes in herbivores can be found in excretory systems, *i.e.* liver, kidney and the gastrointestinal endothelium.[7] In phase I of the elimination process, CYP450 enzymes catalyze oxidation reactions of hydrophobic toxins, leading to reactive hydroxy groups, which can then be used as a "clutch" between the toxins and hydrophilic, water soluble acceptor molecules such as glucose, glucuronic acid, sulfate and glutathione.[67] The latter is described as Phase II metabolism and consists of a conjugation reaction of acceptor molecules with the mentioned agents to ethers and esters, which provides the basis for elimination by the kidney through their high hydrophilic properties.

Likely as a result of various selective advantages during evolution, CYP450 enzymes are subject to numerous egulatory mechanisms. In many cases, substrate molecules act as inducing agents of CYP450 enzymes,[67] *e.g.* 3, 4-benzo(a)pyrene.[68,69] This polycyclic aromatic hydrocarbon is able to activate a specific cytoplasmic receptor, the aryl hydrocarbon receptor (AhR), which, after binding to its ligand and dimerizing with another binding partner, translocates into the nucleus. Here, AhR enhances the expression of CYP1A1 and the metabolism of 3,4-benzpyrene. Polycyclic aromatic hydrocarbons are also responsible for the enhanced expression of phase II genes such as UDP-glucuronosyl transferase and glutathione S-transferase, leading to a strategic connection between phase I and phase II reactions via the so-called "Ah gene battery".[67] Another example of transcriptional induction is the activation of the pregnane x receptor by hyperforin from St John's Wort (*Hypericum perforatum* L.), which causes an increase in CYP3A4 and the drug efflux protein P-glycoprotein. This represents a connection between phase I and phase III reactions for xenobiotic elimination.[7] Furthermore, there is evidence for regulation on the post-transcriptional level via protein stabilization by ethanol, acetone or pyrazole.[67,70] Gender-specific signals also seem to influence CYP450 expression.[71]

The genetic evolution of CYP450 was of immense importance in animal plant warfare because it generated dietary selective pressures responsible for both the development of toxic metabolites in plants and their detoxification in animals.[67] As ancestral CYP genes arose about 3.5 billion years ago, their probable first role was to detoxify oxygen in a reducing atmosphere. Along with the rise of atmospheric oxygen and the shift to an oxidizing atmosphere, a non-linear correlation between the $O_2$ level and the evolution of CYP450 genes took place.[72] About 400 million years ago, several evolutionary forces led to the emergence of many CYP450 families involved in xenobiotic metabolism. At this time, the oxygen level was about 50% of that in the present atmosphere and eukaryotes entering the emerging land masses experienced dietary changes and were exposed to new plant xenobiotics.[73] Furthermore, the atmosphere was enriched in hydrocarbon combustion products. Both sources of toxins necessitated effective detoxification systems, which resulted in new adaptations to changing nutritional sources and environments.[72]

What were the molecular basics of evolution in the CYP gene superfamily? The major force that expedited this development is a phenomenon called "molecular drive," which is a distribution of new gene variants in a population as a consequence of genomic turnover mechanisms.[67] Independent from natural selection and genetic drift,[74,75] this internal irregular DNA behavior may be a major reason for CYP450 evolution. The mechanism of molecular drive is gene duplication, which provides ample opportunity for the establishment of mutations. Whereas one gene copy inherits its original function and must maintain the ability to facilitate necessary cellular processes (a process called 'conservative selection pressure'),[76] the other copy is free to drift, often resulting in a pseudogene, a gene that encodes for a nonfunctional protein. The two copies of the duplicated gene are termed paralogues: related genes arisen by gene duplication. On a molecular level, duplication happens through a polymerase shift. During DNA-replication, the replicated strand may experience a backshift because of repeated elements on the original strand, leading to a loop with duplication of a specific DNA sequence. In contrast, a forward step by the polymerase creates a loop in the original strand, leading to a deletion.[76] The duplicated gene is able to diverge or drift due to mutations. Expedient mutations are fixed, causing evolutionary advantages such as allowing metabolization or synthesis of PSMs.[71] Such evolutionary and counter-evolutionary adaptations have continued in a vicious cycle in enzyme development between plants and herbivores for at least 800 million years. The genes are now considered orthologues, *i.e.* genes in different genomes with a common phylogenetic ancestor.

The evolution of CYP450 by molecular drive can be illustrated by the CYP2D subfamily. About 1.1 billion years ago, CYP2D and other subfamilies emerged though gene duplication events.[71] About 80 million years ago, the split between humans and rodents resulted in marked differences between the enzymatic accoutrements within the two species. While the rat genome contains five active CYP2D genes, human beings possess one active gene and four pseudogenes.[66] The genetic differences between rats and humans are relevant in pharmacology, since results from drug-testing in rodents may be not directly translatable to drug activity in humans.

Caucasian CYP2D6 deficiency is a well-examined genetic polymorphism, which explains why about 10% of the Caucasian population in Europe and North America are unable to efficiently metabolize debrisoquine and other plant alkaloids.[77] The inactivation of *CYP2D6* led to the hypothesis that the gene might be predisposed to extinction, because it rose up during evolutionary animal-plant warfare, but modern civilization requires less dietary alkaloid detoxification.[77]

In general, polymorphisms are common among CYP450s. It is probable that all human CYP450 genes are subject to inter-individual variation, as a result of the enormous genetic diversity of the human species.[72] One reason for the establishment of polymorphisms may be a lack of co-evolutionary pressures. An illustrative example is the CYP2D6 polymorphisms that lead to extensive and poor metabolizers of certain drugs. Extensive metabolizers of debrisoquine show 10-200 times higher metabolite concentration in their urine than poor metabolizers.[72] There is no self-evident advantage for bearing a functionalized gene without drug exposure. In contrast, there is a heightened risk of lung cancer for tobacco smokers who have the extensive CYP2D6 metabolization polymorphism.[66,72]

One approach to measuring these evolutionary incidents are the "per site substitutions per $10^9$ years", meaning the statistical frequency of exchange of a DNA base at a specific site in one billion years. A number of different types of mutations can be defined. Synonymous mutations results in no change in the amino acid sequence encoded, whereas non-synonymous mutations cause an amino acid change and therefore can influence protein structure and function. Silent mutations occur more frequently than do non-silent ones, since they are not subjected to selective pressures. For proteins with strong functional constraints, *e.g.* histones, relatively small numbers of per site substitutions per $10^9$ years (0.004 for non-synonymous and 1.43 for synonym mutations) have been reported. In contrast to these highly conserved genes, the CYP2D6 cluster belongs to a group of rapidly-evolving genes with non-synonymous rates of 1.25 and synonymous rates of 3.91 per site substitutions per $10^9$ years.[77] It is interesting that pseudogenes have similar rates of synonymous and non-synonymous mutations. This can be explained by the fact that selection pressures of evolution do not downsize the number of non-synonymous mutations, because pseudogenes have no function and therefore are free to drift. On the other hand, an active copy is able to inherit the essential function.[77]

## 4 Conclusion and Perspectives

The co-evolutionary adaptation process among higher organisms termed animal plant warfare has resulted in a huge array of bioactive chemical compounds in plants. Because the primary function of these phytochemicals is defense against predators, it comes as no surprise that many of them are toxic to humans. However, a fraction of these plant secondary metabolite compounds exert valuable pharmacological actions and can be exploited for medicinal purposes. Medicinal plant and herbal preparations are frequently advertised as having fewer side effects than chemically synthesized drugs. This claim must be taken with much caution, as beneficial medicinal effects were of course no target for evolutionary selection during the history of animal plant warfare. Therefore, it is an important task of modern pharmaceutical sciences to analyze the molecular and cellular modes of action of plants used for medicinal purposes and to distinguish beneficial pharmacological effects from adverse toxic side effects. This is no trivial task, as plants contain complex mixtures of phytochemicals that interact in the human body, potentially leading to synergistic and/or antagonistic effects. The mechanistic understanding of such interactions may also be exploitable for pharmacological purposes. Plant chemical substances able to inhibit drug transport processes in plants might also serve as inhibitors of human ABC transporters. Drug pumps such as P-glycoprotein (ABCB1/MDR1), MRP1 (ABCC1), BCRP (ABCG2) and others play an important role in physiological processes (*e.g.* in the blood brain barrier) and diseases (*e.g.* in multidrug-resistant tumors). Hence, chemical scaffolds with inhibitory activity towards ABC transporters in plants may be able to serve as lead structures for development of drugs against ABC transporters in human diseases. Other examples are inhibitors of signal transduction molecules (*e.g.* kinase inhibitors) or inhibitors of apoptosis (*e.g.* caspase inhibitors).[78] A deeper understanding of molecular and cellular interactions of phytochemicals and human disease processes opens new avenues for the exploitation of the chemodiversity of plants for drug development.

## Acknowledgments

We thank Karen Duffy (Duke University, Durham, NC, USA) for reading the manuscript.

**Open Access** This article is distributed under the terms of the Creative Commons Attribution License which permits any use, distribution, and reproduction in any medium, provided the original author(s) and source are credited.
